# Do boars compensate for hunting with higher reproductive hormones?

**DOI:** 10.1093/conphys/coab068

**Published:** 2021-09-03

**Authors:** Achiad Davidson, Dan Malkinson, Anat Schonblum, Lee Koren, Uri Shanas

**Affiliations:** 1 Evolutionary and Environmental Biology, University of Haifa, 199 Aba Khoushy Ave., Mount Carmel, Haifa 3498838, Israel; 2 Geography and Environmental Studies, University of Haifa, 199 Aba Khoushy Ave.,Mount Carmel, Haifa 3498838, Israel; 3 Shamir Research Institute, University of Haifa, Qatzrin, 1290000, Israel; 4 Faculty of Life Sciences, Bar-Ilan University, Ramat-Gan, 52900, Israel; 5 Biology and Environment, University of Haifa–Oranim, Tivon, 3600600, Israel

## Abstract

The predation-stress hypothesis has been proposed as a general mechanism to explain the negative effect of predation risk on reproduction, through a chronic activation of the stress response. However, in some cases, stress appears to augment the reproductive potential of mammals. Wild boar (*Sus scrofa*) populations are on a rise worldwide, despite the high hunting pressure that they are exposed to. This hunting pressure instigates, among other effects, earlier sexual maturity in juvenile females, leading to the shortening of wild boars’ generation time. The mechanism that underlies this earlier sexual maturity under high hunting pressure has not been examined to date. To explore the physiological effects that hunting has on the reproductive system and whether the stress response is involved, we examined steroid hormone levels in the hair of female wild boars in northern Israel, comparing populations exposed to high and low hunting pressure. Furthermore, we compared steroid levels in the hair of female wild boars that were roaming alone or as a part of a group. We found no hormonal signs of stress in the hunted boars. Cortisol levels were low in both the high and low hunting-pressure groups. Yet, progesterone levels were higher in females that were exposed to high hunting pressure. Females roaming in a group also had higher progesterone levels compared to females that were alone, with no distinguishable differences in cortisol levels. These elevations in reproductive hormones that were associated with hunting may lead to a higher reproductive potential in female wild boars. They further show that high hunting pressure does not necessarily lead to chronic stress that impairs the reproductive potential of female wild boars. This data suggests that a reproductive hormonal response may be one of the factors leading to the rapid wild boars population growth worldwide, despite the high hunting pressure.

## Teaser

Predation risk usually impairs reproduction, through the stress-response mechanism. However, this study shows that progesterone levels were higher in female wild boars that live in regions of high hunting pressure and roam in a group. Such elevated reproductive hormones, which were associated with high hunting pressure, may lead to a higher reproductive potential in female wild boars.

## 1. Introduction

Prey species commonly minimize predation risk through anti-predator behaviours (Lima and Dill, 1990). These responses usually include changes in vigilance behaviour (Brown, 1999; Lima and Bednekoff, 1999), foraging activity (Brown, 1999; Kotler *et al.*, 1994), space use (Keuling *et al.* 2010; Saïd *et al.*, 2012; Thurfjell *et al.* 2013) and physiology (Bateson and Bradshaw, 1997; Creel *et al.*, 2002; Gobush *et al.*, 2008). These adaptations facilitate an increase in fitness by enhancing immediate survival (Lima, 1998), but they often also incur physiological costs that can affect body condition (Hik 1995) and reproduction (Sheriff *et al.,* 2009; Zanette *et al.,* 2011). The predation-stress hypothesis has been proposed as a general mechanism to explain the negative effects of predation risk on reproduction. The predation-stress hypothesis predicts that encounters with predators affect reproduction and survival through the chronic activation of the stress response (Clinchy *et al.,* 2013). The threat of predation causes an elevation of glucocorticoids (GCs) (Creel *et al.,* 2009; Frid and Dill, 2002; Lima, 1998), which can suppress reproduction (Munck *et al.,* 1984; Romero, 2004; Sapolsky, 2005) through their effects on the hypothalamic–pituitary–gonadal (HPG) axis (Moberg, 1991; Romero, 2004).

The predation-stress hypothesis has been proposed relatively recently and thus has not been studied extensively (Clinchy *et al.*, 2013; Creel *et al.*, 2009; Dulude-de Broin *et al.*, 2020). In recent years, it has received more empirical support (Clinchy *et al.* 2013; Dulude-de Broin *et al.*, 2020; Rey, 2020); however, there is some evidence that in certain systems, the predation-stress hypothesis does not apply. For example, the decrease in the reproduction of elk (*Cervus canadensis*) following the reintroduction of wolf (*Canis lupus*) in Yellowstone National Park (Creel *et al.*, 2007) was mainly tied to constrained foraging activity or efficiency, with no physiological ‘stress-related’ evidence, such as GC elevation (Creel *et al.*, 2009). Moreover, there is some evidence that stress may even elevate reproduction-related hormones in several species (Brandt *et al.*, 2009; Bryan *et al.*, 2015; Cattet *et al.*, 2017). Thus, the ecological conditions under which the predation-stress hypothesis is supported in different species are not fully understood (Creel, 2018; Creel *et al.*, 2009; Dulude-de Broin *et al.*, 2020).

Experimental studies have shown that animals cope with, and respond to, predators partly by activating their hypothalamic–pituitary–adrenal axis, resulting in the release of GC hormones (Boonstra, 2013; Clinchy *et al.*, 2013). Chronic elevation of GCs (i.e. frequently recurring or constant over a long time span) can interrupt the HPG function, whereas short pulses of GC secretion normally do not (Moberg, 1991; Romero, 2004; Sapolsky,2005). Bouts of human hunting may also cause chronic or short-term stress that can result in higher levels of GCs (Bateson and Bradshaw, 1997; Bryan *et al.*, 2015; Creel *et al.*, 2002), particularly if they occur during limited time periods during theyear.

Reproductive hormones can provide additional insight into the effects of hunting on the social structure, behaviour and reproduction of animals. For example, progesterone is elevated in the females of many vertebrate species during pregnancy and the oestrus period and thus can serve as an indicator of long-term population-level reproductive activity (Anderson, 2009; Bryan *et al.*, 2015; Cattet *et al.*, 2017). Furthermore, progesterone in females might be elevated when social conditions are unstable and thus may reflect a stressful social environment (Brandt *et al.*, 2009; Bryan *et al.*, 2013, 2015). However, despite the importance of studying reproductive hormones in the context of conservation and management purposes (Bryan *et al.*, 2015; Creel *et al.*, 2007; Gobush *et al.*, 2008), only a few studies have tested the prolonged effects of hunting on reproductive hormones compared to stress hormones in wildlife (Koren *et al.*, 2019). Moreover, a number of recent studies have revealed that reproductive hormone levels reflect meaningful biological and ecological patterns, such as social and physiological consequences arising from dietary constraints and human hunting (Bryan *et al.*, 2013, 2015; Koren *et al.*, 2019).

Over the past 40 years, the substantial population increases in wild boar (*Sus scrofa*) in agricultural, urban and suburban areas have intensified human–boar conflicts (Linnell *et al.*, 2020; Marsan *et al.*, 1995; Massei *et al.*, 2015). These conflicts have led to elevated economic costs due to disease spillover into livestock and humans, as well as damage to gardens and infrastructure in urban areas and to agricultural crops (Barrios-Garcia and Ballari, 2012; Massei *et al.*, 2015). Hunting is the most common and widespread management tool applied throughout the world to minimize conflicts with wild boars in agricultural areas (Gamelon *et al.*, 2011; Keuling *et al.*, 2013; Massei *et al.*, 2015). A study by Linnell *et al.*, (2020) estimated that more than 3 million wild boars are hunted every year in Europe. Furthermore, the amount of harvested wild boars is constantly rising (Massei *et al.*, 2015). It has been previously shown that such high hunting pressure causes variations to the social structure of wild boar populations (Bieber *et al.*, 2019; Poteaux *et al.*, 2009) and instigates earlier sexual maturity, allowing juvenile females to reproduce earlier (Gamelon *et al.*, 2011; Servanty *et al.*, 2011; Toigo *et al.*, 2008). These consequences eventually causes wild boar generation times to shorten and may eventually lead to the higher reproduction and population growth of wild boars (Servanty *et al.*, 2009; Servanty *et al.*, 2011; Toigo *et al.*, 2008). However, the mechanisms that underlie the shortening of the wild boar generation time under high hunting pressure has not been examined todate.

In this study, we investigated the effects of hunting and social structure on the stress and reproductive hormones of female wild boars in northern Israel. Specifically, we tested cortisol and progesterone levels in the hair of female wild boars roaming alone or as part of a group in areas characterized by high and low hunting pressures. It has already been demonstrated that females tend to roam in bigger groups when predation risk is high, most likely because it provides them with a higher sense of security due to greater chances of predator detection (Pays *et al.*, 2012; Podgórski *et al.*, 2016; Roberts, 1996). Thus, we hypothesized that female wild boars roaming in a group would have lower stress hormones, and accordingly higher reproductive hormones, compared to solitary wild boars. Moreover, we hypothesized that in areas of high hunting pressure, female wild boars would have higher levels of stress hormones, and accordingly lower reproductive hormones, compared to areas of low hunting pressure.

## 2. Materials and methods

### 2.1. The study area

**Figure 1:**
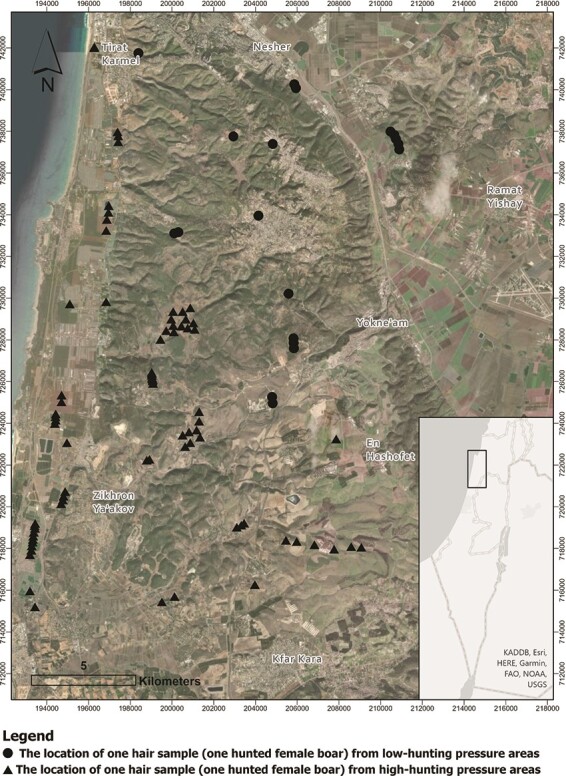
The study area in the Carmel coastal mountain range, Israel, including the high and low hunting pressure agricultural areas where hair samples were collected.

The study took place in the Carmel coastal mountain range in northern Israel (Fig. 1), an area of ~600 km^2^, with an elevation ranging from 0 to 546 m above sea level. The climate is Mediterranean, and the annual rainfall varies between 500 and 700 mm, falling mainly (80%) from December to March. The natural vegetation is a typical Mediterranean maquis (Hadar *et al.*, 1999; Neeman *et al.*, 1995), intermixed with cultivated areas.

Within the study area, the main predator of wild boars in Israel, the wolf, has been considered as locally extinct. However, there are other mortality-causing factors, such as roadkill and especially hunting. To the best of our knowledge (given the ample food availability in the agricultural areas) roadkill and especially hunting are the dominant exogenous stress factors in the study site. This region exhibits the highest hunting pressure in Israel, as well as the country’s highest reported number of human–wild boar conflicts in agricultural landscapes (Lider, 2012) and urban areas (Malkinson, 2015). Hunting is prohibited in nature reserves and authorized in agricultural areas (given the required permits) throughout the year; there are no hunting seasons. To account for hunting pressure in the studied region, the agricultural areas were divided into polygons and each was assigned a hunting pressure from 1 (low) to 3 (high). Polygons were not identically delimited throughout the study area. Polygons were delineated according to estimated hunting regimes in the different areas and were mainly marked around villages or towns of different sizes and shapes. Furthermore, polygons with different hunting pressures were far enough from each other in order to minimize the probability that individuals did not move among them. Namely, the distance between polygons were much greater than the daily home-range sizes of wild boars in rural areas (1–1.6 km^2^) (Boitani *et al.*, 1994; Morelle *et al.*, 2015; Russo *et al.*, 1997). Because the Israel nature and parks authority (INPA) generally does not formally record the exact location of hunting events, we used the expert opinions of three different INPA ecologists and rangers and cross-validated them with local hunters’ expertise to estimate the hunting pressure within each polygon. Then all polygons that were assigned with the values of two and under were categorized as low hunting-pressure areas and polygons that were assigned with values greater than 2 were categorized as high hunting-pressure areas. Hof HaCarmel Regional Council is the only area within the study site that the INPA thoroughly records hunting events. Thus, we used Hof HaCarmel hunting records as a reference for the rest of the polygons in the study site. The hunting pressure in Hof HaCarmel was assigned by the INPA ecologists and rangers with a value of 3, based on an average of 40 hunting campaigns/week that are carried out throughout the year. On average, 870 boars/year are hunted in Hof HaCarmel, which has a jurisdiction of 190km^2^. Other regions that were assigned with the value of 3 have similar hunting regimes. In low hunting pressure areas INPA rangers estimate that the average number of hunting campaigns is approximately 3 per week throughout the year. Despite differences in hunting regimes, habitats are quite similar throughout high and low hunting pressure areas—they both have agriculture areas in valleys and agriculture areas in mountainous regions (Fig. 1). In both high and low hunting pressure areas, wild boars hide during the day in the dense Mediterranean maquis vegetation, and during the night they forage in the nearby agricultural areas. There are no official estimates of wild boars densities in the study region, or anywhere else in Israel (except for the average amount of hunted boars per year from Hof HaCarmel).

### 2.2. Hair sample collection

Mammalian hair, which integrates steroid hormones as it grows, can provide a valuable resource to investigate physiological responses to natural processes and potentially prolonged stressors, such as hunting (Bryan *et al.*, 2015; Macbeth *et al.*, 2010; Meyer and Novak, 2012). Levels of hormones in hair have been demonstrated to be correlated with measures in blood, saliva and faeces in several mammalian species (Accorsi *et al.*, 2008; Bennett and Hayssen, 2010; Morgan *et al.*, 2019). However, compared with other noninvasive sampling methods, the investigation of hair has several features that make this method advantageous or complementary to assess hormone levels (Gormally and Romero, 2020). For example, although steroids are often excreted as metabolites in urine and faeces, steroids remain intact in hair (Koren *et al.*, 2019). Furthermore, steroid hormones in hair appear to be stable for months to years or more (Macbeth *et al.*, 2010). Thus, unlike other steroid sampling techniques, steroid hormones in hair present opportunities to study the prolonged effects of stressors, such as hunting (Bryan *et al.*, 2015; Gormally and Romero, 2020; Koren *et al.*, 2019).

We provided paper envelopes to INPA rangers and expert hunters, who then returned them with hair samples of hunted wild boars enclosed. All samples were collected from fresh carcasses immediately after they were shot. The hair was cut from the hindquarters (posterior) of the wild boars with scissors, as closely as possible to the root. Tufts of hair (20–5170 mg) were placed in dry paper envelopes and kept at room temperature for up to 2 years before hormonal assays were performed (Bryan *et al.*, 2015). The rangers and hunters provided the following information with each sample envelope: the date and location of the hunting event, the age and sex of the hunted boar and whether the hunted individual was alone or a part of a group (Tables 1, 2). The hair samples were collected between 2016 and 2018 as part of an ongoing control program to mitigate wild boar damages to crops in agricultural areas. Due to an insufficient number of samples, we excluded the winter season from our seasonal analysis of the wild boars’ hormones. For each hair sample location, we assessed stress and reproductive hormone levels as dependent on the season, level of hunting pressure and social structure (Tables 1, 2).

**Table 1:** Summary of the number of samples collected and analysed and the available information regarding season and social structure of the samples

	Number of samples	Available seasonal data	Available social structure data
Progesterone	95	62	48
Cortisol	133	100	55

**Table 2:** Sample sizes of females in group and solitary females

	Females in group	Solitary females
High hunting pressure	32	5
Low hunting pressure	7	4

In the northern hemisphere, mammalian winter coats are composed of longer and denser hair than summer coats (Berman and Volcani, 1961; Mowafy and Cassens, 1976). It had been shown that the hair of domesticated pigs (*Sus scrofa domesticus*), in Wisconsin grew 54 mm in ~5 months during spring to autumn (Mowafy and Cassens, 1976). However, we do not have information about hair growth of wild boars in Israel (or other Mediterranean countries). Nevertheless, we do know that other mammals in Israel show continuous hair growth throughout the year (Koren *et al.*, 2019b).

### 2.3. Measurements of steroids in hair

In the laboratory, we extracted and quantified steroid hormones from female wild boar using standard protocols (Fishman *et al.*, 2018). The mass of the samples varied depending on the availability. The entire hair in the taft was used for the steroid analysis. All hair samples were carefully weighed, and the mass was recorded, and then cut into smaller pieces and placed in a Petri dish (10 × 20 mm or 90 × 15 mm; De-groot Ltd, Rosh Haayin, Israel). Briefly, we washed hair samples twice with isopropanol (Romical Ltd, Beer Sheva, Israel), while we mixed them on an orbital rotator for 3 minutes. Next, we dried the samples for 12 hours, and then cut the hair into smaller pieces so that it would fit into the vials (20 ml, Yoel Naim Ltd, Rehovot, Israel). We added 2 ml of methanol (Sigma-Aldrich Israel Ltd, Rehovot, Israel), and then the sample was sonicated for 30 minutes (MRC, model DC150H), followed by incubation overnight at 50°C while shaking. The next day, after the vial had cooled to room temperature, we transferred the methanol and steroids to a polypropylene Eppendorf tube (De-groot Ltd, Rosh Haayin, Israel), and centrifuged it in order to separate them from unwanted particles (Thermo Scientific, model microCL 178R) for 10 minutes at 13.3 RPM at 4°C. Then the methanol was transferred to a glass vial and evaporated under a stream of nitrogen at 45°C using a Techne Sample Concentrator (FSC496D). Samples were reconstituted in 10% methanol and 90% assay buffer (provided by the kit manufacturer), and steroids were quantified using commercial competitive Enzyme-Linked Immunosorbent Assay (ELISA; Salimetrics Europe, Newmarket, for cortisol and for progesterone, UK) kit. For progesterone, the manufacturer reported that antibody cross reactivity with other steroids was less than 0.192%. For cortisol, it was reported that antibody cross-reactivity with dexamethasone was 19.2% and <0.568% with all other steroids. Cortisol and progesterone were validated for female wild boar hair by conducting serial dilutions of separate hair pools, consisting of more than 12 random samples and testing for linearity (10–350 mg and 0.5–2 mg, respectively) and parallelism (slope covariance, *P =* 0.641 and *P =* 0.361, respectively) with the kit standards provided. Intra-assay variability (CV) was 4.5% for cortisol and 9.1% for progesterone for 6 repetitions of the pool on the same plate. Inter-assay CV was 10.2% for cortisol and 10.09% for progesterone across 5 plates. Recovery was 90% for cortisol and 102.2% for progesterone, with results quantified by comparing hair samples spiked with a known amount of cortisol or progesterone to unspiked samples.

### 2.4. Statistical analysis of hormonal data

Data on cortisol and progesterone and concentrations were log transformed to achieve normality. Model assumptions of equality of variances (Levene’s test) and normality of residuals (Kolmogorov–Smirnov test) were met. For both cortisol and progesterone, we used a two-way analysis of variance (ANOVA) to assess interactions between hunting pressure and social structure. We applied Tukey’s honestly significant difference (HSD) tests post hoc. Furthermore, we used *t*-tests to compare differences in the cortisol and progesterone of female wild boars, between high and low hunting pressure areas.

**Table 3:** Coefficients and goodness-of-fit measure of the two-way ANOVA linear model relating progesterone levels to hunting pressure and social structure

Coefficient	Estimate	Std. error	*t*-value	*P*-value	*R^2^*
					0.28
Intercept	1.105	0.047	23.256	<0.001^*^	
Hunting pressure	−0.307	0.097	−3.151	0.002^*^	
Social structure	−0.248	0.105	−2.364	0.022^*^	

Results are based on the social structure data set (48 hunted adult female wild boars).

**Figure 2:**
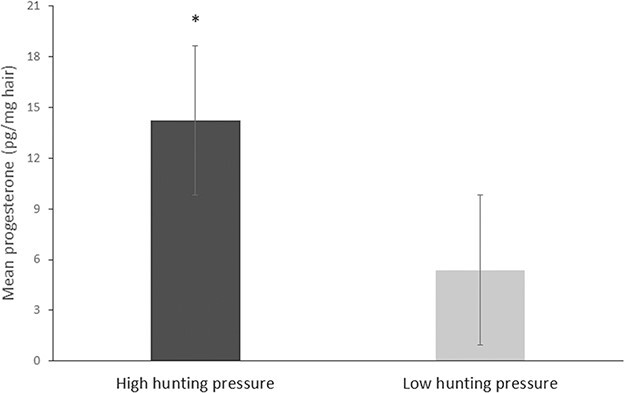
Mean ± SE hair progesterone concentrations (picograms per milligrame of hair) collected from 48 hunted adult female wild boars (social structure data set) in high and low hunting pressure areas (high, *n* = 37; low, *n* = 11). The asterisk denotes significant differences among populations (*P* = 0.001).

## 3. Results

### 3.1. Evaluating cortisol and progesterone levels of female wild boars

The mean cortisol was $\overline{x}$ =2.0 pg/mg, *SD* = 1.6. Season (*F*_*2*,97_ = 1.40, *P* = 0.250) and hunting (*t*_(24.77)_ = 0.71, *P* = 0.483) did not have a significant effect on hair cortisol levels. Group structure did not have a significant effect on cortisol levels (*t*_(11.03)_ = −0.02, *P* = 0.984). The mean progesterone was $\overline{x}$ =10.2 pg/mg, *SD* = 9.5. Season had a significant effect on hair progesterone levels (*F*_2,59_ = 3.31, *P* = 0.043), but a post hoc Tukey’s HSD analysis revealed no significant interactions among the different seasons: spring–autumn (adjusted *P* = 0.930), summer–autumn (adjusted *P* = 0.114) and summer–spring (adjusted *P* = 0.104). Land use (agriculture areas in valleys or Mount Carmel) did not have a significant effect on cortisol (*t*_(17.64)_ = −1.04, *P* = 0.308) or progesterone (*t*_(5.77)_ = 1.59, *P* = 0.163). There was no significant correlation between cortisol and progesterone r_(93)_ = 0.15, *P* = 0.127.

### 3.2. The effect of hunting and social structure on progesterone levels

**Figure 3:**
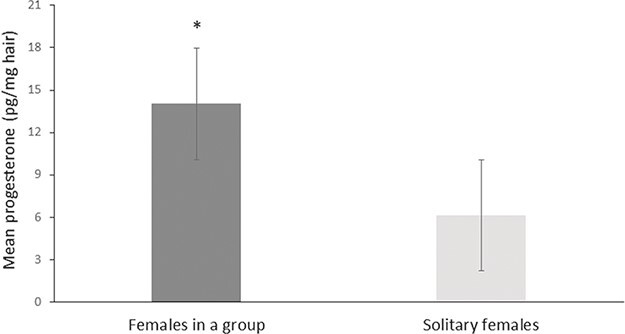
Mean ± SE hair progesterone concentrations (picograms per milligrame of hair) collected from 48 hunted adult female wild boars (social structure data set) that roamed as a part of a group or alone (group, *n* = 39; solitary, *n* = 9). The asterisk denotes significant differences among populations (*P* = 0.026).

A two-way ANOVA showed that both hunting pressure (*F*_1,44_ = 14.57, *P* = 0.001) and social structure (*F*_1,44_ = 5.49, *P* = 0.023) had a significant effect on the progesterone levels of female wild boars (adjusted *R^2^* = 0.28; Table 3). Although both hunting and social structure had a significant and additive effect on progesterone levels, there was no interaction between them (F1,44 = 0.228, *P* = 0.63; sample sizes are presented in Table 2). A post hoc Tukey’s HSD analysis of the social structure data set indicated that adult females that were exposed to high hunting pressure had significantly higher progesterone levels compared to females exposed to low hunting pressure (*P* = 0.001; Fig. 2). Furthermore, adult females that roamed as part of a group had significantly higher progesterone levels compared with solitary females (*P* = 0.026; Fig. 3). In order to evaluate the relative effect of the estimates of social structure (−0.248; Table 3) and hunting (−0.307; Table 3), we standardized the values of the coefficients. The ratio between the standardized estimates of social structure and hunting was 0.874. This result indicates that hunting and social structure had almost the same relative effect on progesterone levels, with hunting slightly more influencing.

**Figure 4:**
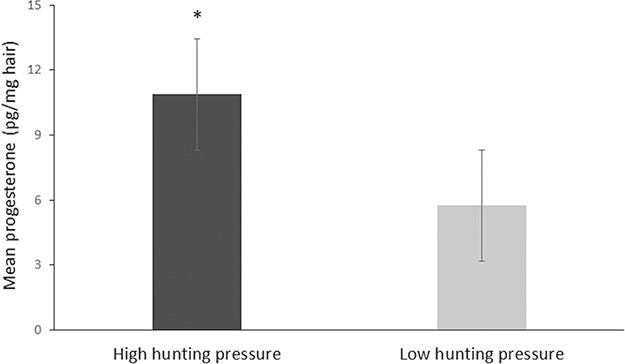
Mean ± SE hair progesterone concentrations (picograms per milligrame of hair) collected from 95 hunted adult female wild boars (full data set) in high and low hunting pressure areas (high, *n* = 78; low, *n* = 17). The asterisk denotes significant differences among populations (*P* = 0.01).

In order to complement the two-way ANOVA, and further evaluate the effect of hunting on progesterone levels with a bigger sample size, we also analysed the effect of hunting on the progesterone levels of 95 adult females (Table 1). We found that females exposed to high hunting pressure had significantly higher progesterone levels compared to females exposed to low hunting pressure (*t*_(42.51)_ = 2.66, *P* = 0.010; Fig. 4). The results revealed no significant differences in progesterone levels among the different seasons. The average progesterone levels, however, were higher in the high hunting pressure areas compared to the low hunting pressure areas (Appendix 1).

## 4. Discussion

Contrary to the predation-stress hypothesis and to our prediction, we did not find higher GC levels in female wild boars that were exposed to high hunting pressure. However, these females had higher progesterone levels, which may reflect higher reproductive efforts as a result of exposure to high hunting pressure (Bryan *et al.*, 2015). Additionally, we found that females in groups had significantly higher progesterone levels compared to solitary ones. Our results show an additive effect of hunting and social structure on the levels of reproductive hormones of female wild boars. Thus, the significantly higher progesterone levels we detected in female wild boars in high hunting pressure areas may be linked to the social disruption caused by high hunting pressure (Bieber *et al.*, 2019; Massei *et al.*, 2015; Poteaux *et al.*, 2009).

Turner and Tilbrook (2006) suggested that cortisol levels need to be elevated in a sustained manner for a substantial period (>4 days) in female domesticated pigs before reproduction is negatively affected and, even then, reproduction in some individuals appears to be resistant to its effects. Moreover, in recent years, evidence has accumulated on the positive role of short elevations in stress on the luteinizing hormone (LH) secretion and ovarian function of female pigs (Madej *et al.*, 2009; Turner and Tilbrook, 2006; von Borell *et al.*, 2007). This LH secretion forms the basic conditions for the activation of ovaries in terms of follicular growth, maturation and ovulation (Stančić *et al.*, 2012). For example, Paterson and Pearce (1989) found that female pigs that were exposed to short-term stress were more responsive to puberty stimulation and reached puberty earlier compared to ‘gently’ handled gilts. Furthermore, Brandt *et al.* (2009) found that cortisol concentrations that were elevated in the short term significantly increased progesterone concentrations in female pigs. Because domesticated pigs are essentially the same species as wild boars (*S. scrofa*), it is expected that short-term stress can also enhance reproductive hormones, fertility and sexual puberty in wild boar populations.

Wild boars are exposed to high hunting pressure worldwide (Linnell *et al.*, 2020; Massei *et al.*, 2015). It has been previously demonstrated that this high hunting pressure significantly affects their behaviour; for instance, their dispersal patterns (Keuling *et al.*, 2010) and activity and spatial-usage patterns (Keuling *et al.*, 2008; Scillitani *et al.*, 2010; Thurfjell *et al.*, 2013). Furthermore, hunting may also cause wild boars to select safer habitats, even at the expense of decreased access to resources (Saïd *et al.*, 2012). Moreover, our unpublished research shows that hunting causes higher levels of vigilance behaviour in wild boars in general, and in females in particular (Davidson *et al.* 2021, in review). Thus, it is expected that these behavioural responses are caused by stress induced by hunting, among other factors. In the wild, it has been demonstrated that female wolves exposed to high hunting pressure have elevated progesterone and cortisol levels (Bryan *et al.*, 2015). Bryan *et al.* (2015) suggested that this increase in stress and reproductive hormones may reflect an increased reproductive effort in response to hunting. As mentioned above, short-term stressors often do not have a negative effect and even may stimulate reproduction and enhance fertility of female pigs (Madej *et al.*, 2009; Turner and Tilbrook, 2006; von Borell *et al.*, 2007). Thus, we suggest that repeated bouts of short-term stress caused by hunting campaigns may also stimulate higher levels of reproductive hormones in female wild boars.

Contrary to our prediction at the outset of the study, there was no difference in cortisol levels between female wild boars roaming in a group or alone. However, females roaming in a group did show significantly higher progesterone levels compared to solitary female wild boars. The proportion of time spent engaging in vigilant behaviour is associated with both short- and long-term stressors (Morgan and Tromborg, 2007), and factors such as group size and habitat characteristics can influence it (Chmura *et al.*, 2016). In many species, including wild boars (Podgórski *et al.*, 2016; Quenette and, Gerard 1992), individuals tend to decrease their vigilance behaviour by increasing their group size (Pays *et al.*, 2007, 2012; Roberts, 1996). Bigger groups provide individuals with a higher sense of security due to greater chances of predator detection and fewer chances of been preyed upon (Pays *et al.*. 2012; Podgórski *et al.*. 2016; Roberts, 1996). Furthermore, Saïd *et al.* (2012) and Scillitani *et al.* (2010) both found that hunting caused females with offspring to change their activity and spatial usage significantly more than males. These differences between sexes might result from females with offspring responding more strongly to hunting (Saïd *et al.*, 2012). Thus, it is expected that in high hunting pressure areas, breeding females (with relatively high progesterone levels) with offspring will prefer to breed and raise their young in larger groups of females, as opposed to nonbreeding females (with relatively lower progesterone levels) with no offspring. This suggests a combined effect of social structure and hunting.

Furthermore, the mortality among individuals, especially adults, due to hunting has been considered a potential driver of variations in the social organization of wild boar populations. These variations may lead to the disassembly of family groups and thus to a chaotic social structure; disorientation among the remaining yearlings of the group may affect their social status and eventually reproduction (Bieber *et al.*, 2019; Keuling *et al.*, 2010; Poteaux *et al.*, 2009). Additionally, it had been shown that hunting may facilitate the breakup of wild boars’ polygynous mating system, due to selective hunting of adult males, and thus may contribute to a higher number of males in the next generation and the early access to reproduction for young males, even within the same social group (Poteaux *et al.*, 2009). The consequent effects of hunting for social structure, social status and breeding strategies of wild boars may enhance the progesterone levels of female wild boars. Progesterone levels in female pigs are significantly elevated during the rut season (estrus) and pregnancy (Anderson, 2009). Thus, the significantly elevated progesterone levels that we found in females that were exposed to high hunting pressure may indicate higher reproductive potential. Despite this, to the best of our knowledge there is no evidence of higher reproductive success in areas with higher hunting in our study site. Thus, we suggest that elevations in reproductive hormones that were found to be associated with hunting may lead to a higher reproductive potential (and not necessarily to reproductive success). There are many factors that affect reproductive success in rural areas. Thus, it is hard to make a direct link in wild populations between reproductive potential and success. For instance, we may not see an increased prevalence of juveniles in high hunting pressure areas because a lot of juveniles are being hunted in these areas.

The accumulated evidence from the literature together with our findings on the effects of hunting on the behaviour and social structure of wild boars suggest that hunting stress combined with a disrupted social structure may have an additive effect on the progesterone levels of female wild boars (Bieber *et al.*, 2019; Bryan *et al.*, 2015; Poteaux *et al.*, 2009). Furthermore, our results indicate that hunting and social structure had almost equal additive effects on progesterone levels. Despite this, we did not find that hunting had a significant effect on the cortisol levels in the hair of female wild boars. Possibly, short bouts of GCs do not appear in wild boar hair, because they are quickly removed from the body. Additionally, cognitive and emotional aspects of avoiding predation are still unknown, thus predation risk effects may also occur through mechanisms that do not involve the stress response (Creel *et al.*, 2009). Moreover, our results suggest that there are probably other factors affecting progesterone levels that were not tested in our research, such as sex ratios, genetic structure and other social factors that may affect wild boars’ reproduction across several generations. Thus, we encourage further studies that will explore the possibility that other behavioural, social, genetic and reproductive factors may affect GC and progesterone levels of female wild boars.

Nevertheless, our findings highlight the importance of studying reproductive hormones for management purposes (Bryan *et al.*, 2013, 2015; Gobush *et al.*, 2008), especially because few studies have tested the prolonged effects of hunting on the reproductive hormones found in the hair of wildlife (Koren *et al.*, 2019). In conclusion, our study suggests that elevated reproductive hormones, which were associated with high hunting pressure, may lead to a higher reproductive potential in female wild boars. Furthermore, our study provides evidence that increased predation risk does not necessarily lead to chronic stress that impairs the reproductive potential of female wild boars. This response may be one of the reasons leading to the worldwide rapid population growth of wild boars, despite the high hunting pressure they are exposedto.

## Funding

This work was supported by a research grant provided by the Rothschild Foundation Hanadiv Europe.

## Supplementary Material

Appendix_1_coab068
