# Supplementary material for: Do boars compensate for hunting with higher reproductive hormones?
Source: Conserv Physiol. 2021 Sep 3;9(1):coab068. doi: 10.1093/conphys/coab068 (PMC8415534; doi:10.1093/conphys/coab068)
Supplement: Appendix_1_coab068 [file appendix_1_coab068.docx]

**SUPPLEMENTARY MATERIAL**

**Appendix 1:** Mean values ± SD (*n*) of hair progesterone (picograms per milligram of hair) according to the different seasons and hunting pressures.

|  | High hunting pressure | Low hunting pressure |
| --- | --- | --- |
| Autumn | 7.5 ± 5.0 (*n*=10) | 6.9 ± 3.4 (*n*=7) |
| Spring | 6.7 ± 2.2 (*n*=7) | 4.1 ± 1.6 (*n*=3) |
| Summer | 11.1 ± 10.0 (*n*=28) | 6.1 ± 2.5 (*n*=7) |
